# An outbreak of epidemic keratoconjunctivitis caused by human adenovirus type 8 in primary school, southwest China

**DOI:** 10.1186/s12879-019-4232-8

**Published:** 2019-07-15

**Authors:** Duo Li, Jie-Nan Zhou, Hong Li, Cun-Ying He, Qing-Shan Dai, Xiang-Lan Li, Jian-Fang He, Hong He, Ming-Bao Li, L I-Li Jiang, Yao-Yao Chen, Wen Xu

**Affiliations:** 1 Yunnan Provincial Center for Disease Control and Prevention, Kunming, Yunnan People’s Republic of China; 2 Diqing Tibetan Autonomous Prefecture Center for Disease Control and Prevention, Shangri-La City, Yunnan People’s Republic of China; 3 Weixi Lisu Autonomous County Center for Disease Control and Prevention, Weixi Lisu Autonomous County, Diqing Tibetan Autonomous Prefecture, Yunnan People’s Republic of China

**Keywords:** Human adenovirus 8, Epidemic Keratoconjunctivitis, Risk factors, Phylogenetic

## Abstract

**Background:**

Two outbreaks of epidemic keratoconjunctivitis (EKC) occurred successively with an interval of 5 days in two primary boarding schools in Weixi Lisu Autonomous County, Diqing, and Tibetan Autonomous Prefecture, Yunnan. The aims of this study were to determine the intensity and characteristics of the outbreaks, as well as the clinical manifestations in the patients, the risk factors for infection and the pathogen responsible for the two outbreaks.

**Methods:**

An outbreak investigation was conducted in two primary schools, and a case-control study including patients from the Weixi County Ethnic Primary School was performed. Relevant specimens were collected according to the case definition, and next-generation sequencing was employed to identify the pathogen. An epidemiological investigation method was used to analyse the related epidemiological characteristics, such as risk factors. The phylogenetic tree was constructed by MEGA 7.0.

**Results:**

A total of 331 acute conjunctivitis cases, including probable cases of EKC, were reported in the two schools, and the attack rates were 30.59% (171/559, 95%*CI*: 26.76–34.42) and 20.41% (160/784, 95%*CI*: 17.58–23.24), respectively. Cases occurred in all grades and classes in both schools, and only one staff member in each school presented illness. The epidemics lasted for 54 days and 45 days, respectively. The patients had typical manifestations of EKC, such as acute onset, follicular hyperplasia, pseudomembrane formation, preauricular lymphadenopathy, corneal involvement and blurred vision, and a relatively long disease course (average 9.40 days, longest 23 days and shortest 7 days). The risk factor for infection was close contact with a patient or personal items contaminated by a patient. The pathogen responsible for the outbreaks was HAdV-8. The virus was highly similar to the 2016 HAdV-8 strain from Tibet, China.

**Conclusions:**

This study strongly suggests that HAdV-8 could lead to serious consequences. This is the second report of a HAdV-8-associated EKC outbreak in mainland China. Tibetan HAdV-8 might be circulating in southwest China; therefore, it is necessary to monitor the pathogens causing acute conjunctivitis in this area.

## Background

Acute infectious conjunctivitis is a very common disease that is usually caused by viruses or bacteria; it has high incidence rates. Outbreaks of acute infectious conjunctivitis can occur in schools, military camps and hospitals where people gather, and it can impose economic and social burdens [1, 2]. In recent years, viral conjunctivitis has been the most common form, followed by bacterial conjunctivitis [3, 4]; adenoviruses are one of the main causes of viral conjunctivitis. Human adenoviruses (HAdVs) are non-enveloped, double-stranded DNA viruses [5–11] with icosahedral capsids in the family Adenoviridae. The viral particles, which are approximately 65 to 80 nm in size, consist of protein capsids, core proteins and DNA. The HAdV capsid consists of seven structural proteins, three major capsid proteins, hexon, fibre, penton, and four minor ‘cement’ proteins, namely, protein IIIa (pIIIa), VI, VIII and IX (pIX). Among them, hexon, as a major capsid component, is a target for host immune responses against HAdV, Fibre and penton are important for viral cell entry, as they bind to cellular receptor(s) [12]. Based on their serological, biochemical and genetic properties, HAdVs have been classified into seven species (A-G) [11] and 88 types. More than 10 types of HAdVs are associated with common ocular infections, and HAdV 1–5, 7, 8, 11, 14, 19, 22, 37, 42, 48, 53, 54, 56, and 64 are associated with acute conjunctivitis. Of these, HAdV-8, − 19 and − 37 cause a severe form of epidemic keratoconjunctivitis (EKC) [5, 7–9, 13–17]. In addition, EKC [18–20] is caused by enterovirus type 70, human coxsackievirus A24 variant (CVA24v), and HAdV-11. Outbreaks caused by adenovirus types 8, 56, 54 and enteroviruses CVA24v and EV70 have been reported in China and other countries or regions around the world [6, 15, 21–27].

In May 2018, two outbreaks of EKC occurred in two primary schools in Weixi Lisu Autonomous County, Diqing Tibetan Autonomous Prefecture, Yunnan Province. We conducted an outbreak investigation to identify the pathogen causing the two outbreaks; identified its genetic characteristics to analyse the intensity and characteristics of the outbreaks, the clinical manifestations of the patients, and the risk factors associated with acute conjunctivitis transmission within the schools; and provided infection control recommendations.

## Methods

### Study area

Diqing Tibetan Autonomous Prefecture is located in northwest Yunnan Province and borders the Tibetan autonomous region. Weixi Lisu Autonomous County is one of the counties under the jurisdiction of Diqing Tibetan Autonomous Prefecture.

### Outbreak information

In May 2018, two outbreaks of acute conjunctivitis occurred in Weixi County Ethnic Primary School and Yongchun Township Central Primary School in succession. Both schools were full-time boarding schools; the students returned home on weekends. Nine to 11 students shared a dormitory in both schools. The students used their own face towels, soap, toothpaste, cups, and basins. Sometimes, some students shared soap with others. Students in the same dormitory shared a towel hanging rack. The students washed their faces, brushed their teeth and bathed in a public area. The toilets in the schools flushed automatically. The first outbreak appeared in the County Ethnic Primary School. There were 559 teachers and students in the school, including 515 students (male 263, female 252) and 44 staff. Grades 4 to 6 contained 12 classes. Yongchun Township Centre Primary School was 13 km from the county township. The school had 784 teachers and students. Among them, there were 730 students (male 377, female 353) and 54 staff. There were 19 classes in grade one to six (Table 1).

**Table 1:** Basic information of outbreaks in two schools, 2018

Variables	County ethnic primary school	Township central primary school	*P* value
Number of students (staff) in school	515(44)	730(54)	
Number of cases among students (staff)	170(1)	159(1)	
Number of classes (grades) in school	12(3)	19(6)	
Number of classes (grades) case occurred	12(3)	19(6)	
Average (max, min) number of cases in class	14.17(29,2)	8.32(20,1)	
Date of onset for index case (date/month)	18/5	23/5	
Date of onset for last case (date/month)	10/7	6/7	
Epidemic duration (day)	54	45	
Number of cases (attack rate %)	171(30.59)	160(20.41)	<0.001
Disease course (day)- average (max, min)	–	9.40(7, 23)	
Characteristic of cases			
Gender (%)			<0.001
male	89(52.05)	117(73.12)	
female	82(47.95)	43(26.88)	
Age- median (max, min)	12.34(9.91, 29.53)	11.00(7, 37)	

### Case definition

An EKC case was defined as any clinically suspected case of conjunctivitis, characterized by a redness of the eye with symptoms that included pain, itching, or a foreign body sensation accompanied by tearing or discharge that occurred on or after May 6, 2018, and May 11, 2018, in the County Ethnic Primary School and Yongchun Township Central Primary School, respectively.

### Outbreak investigation

A field investigation was conducted in accordance with the guidelines for field epidemiology [28]. We randomly selected one of the schools experiencing an outbreak to conduct case-identification and case-control studies with face-to-face interviews using a structured questionnaire administered by trained investigators; the Ethnic Primary School was chosen. The variables in the questionnaire for case-identification included essential information such as name, sex, age, onset date, grade, class, and clinical manifestations. An ophthalmologist inquired about the patient’s symptoms, examined the patient with a slit lamp, and made a clinical diagnosis. We adopted a 1:1 case-control methodology. According to a bilateral test level of *α* = 0.05, the power of test 1-*β* = 0.90 and an exposure rate of the control group of *P*_*0*_ = 0.178, *OR* = 3.38 [29], as obtained from the literature, the sample size was determined at 71 cases and 71 controls by formula calculation.

In the case-control study, many variables were considered as infection risk factors based on living habits of the students in the school as well as previous studies. These variables included the number of individuals with acute conjunctivitis in their family, in the same class and in the same dormitory; the frequency of contact with the patient’s eyes or hands or articles used by the patient; the frequency of sharing face towels, washbasins, soap, bedding, pillows, water cups, eye drops, and thermoses with patients; the number of shared water dispensers in classrooms and dormitories; the frequency of eye rubbing per day; the toilet type and faucet type; the frequency of wiping sweat with their hands; and the frequency of washing their hands before meals and after using the toilet.

A case-control study was conducted in the County Ethnic Primary School. All cases in grade 4–5 were included; those in grade 6 were not included, as students were taking a graduation examination. The corresponding control group was selected using simple random sampling from a list of student names in the same class with the IBM SPSS Statistics 25.0 software package (IBM Corp, Armonk, NY, USA). In cases when the sample in the class was insufficient, more samples were obtained from a neighbouring class using the same method.

### Specimen and data collection

During the EKC outbreaks in the Ethnic Primary County and Yongchun Township Central Primary Schools, a total of 38 conjunctival swab specimens were collected from 38 patients with symptoms such as conjunctival congestion and hyperaemia during the acute phase of infection by epidemiology staff of the Diqing Center for Disease Control and Prevention (CDC). After aseptic collection, specimens were transferred to test tubes containing 2 ml minimal essential medium (MEM). Specimens were stored at 4 °C and delivered to the Yunnan Provincial Center for Disease Control (YNCDC), maintaining the cold chain, within 24 h for further investigation.

### DNA extraction and PCR amplification

Viral nucleic acids of conjunctival swabs were extracted using a QIAamp Viral RNA Mini Kit (Qiagen, Hilden, Germany) and QIAamp DNA Mini Kit (Qiagen, Hilden, Germany). Labelled probes were applied to detect enteroviruses, adenoviruses and chlamydia by real-time PCR (Bio-Rad CFX96, California, USA). The primers and probes were included in the commercial Rapid Detection of Respiratory Pathogens Kit (Shenzhen United Medical Technology Co., Shenzhen, China). The experimental operation followed the instructions in the manual.

### Cell culture and virus isolation

Virus isolation from HAdV-positive samples was performed using HEp-2 cells according to standard procedures [30]. Cytopathic effects (CPEs) were observed daily, and the virus was harvested when > 70% cells had developed adenovirus-like CPEs within 7 days. If CPEs were not observed within 7 days of incubation, two extra passages were performed.

### Next-generation sequencing and sequence analysis

A MiSeq sequencing library was prepared for deep sequencing using an Illumina Nextera XT Kit. The sequencing results were analysed by the CLC Genomics Workbench 9.5.2 (Qiagen, Denmark) and submitted to GenBank (isolation ID: MH 634393). The sequence was aligned by using MEGA software (version7.0) with the maximum likelihood method to construct the phylogenetic tree of the whole adenovirus genome and three major capsid genes, hexon, fibre and penton. The guiding value was 1000 (other parameters were default values).

### Statistical analysis

Data analysis was performed with R software (version 3.4.4). The characteristics of the cases were described in terms of frequency, percentage, median, maximum, and minimum. Risk factors for infection were analysed in the univariate analysis using the chi-square test. Finally, logistic regression predictions were performed on the variables with significant differences to correct for other possible confounders. The final significance level was 0.05.

### Ethics statement

The patient’s relevant information and the collection of conjunctival swab specimens were obtained with informed consent from the patients and the school authorities.

## Results

### Outbreak investigation

**Fig. 1:**
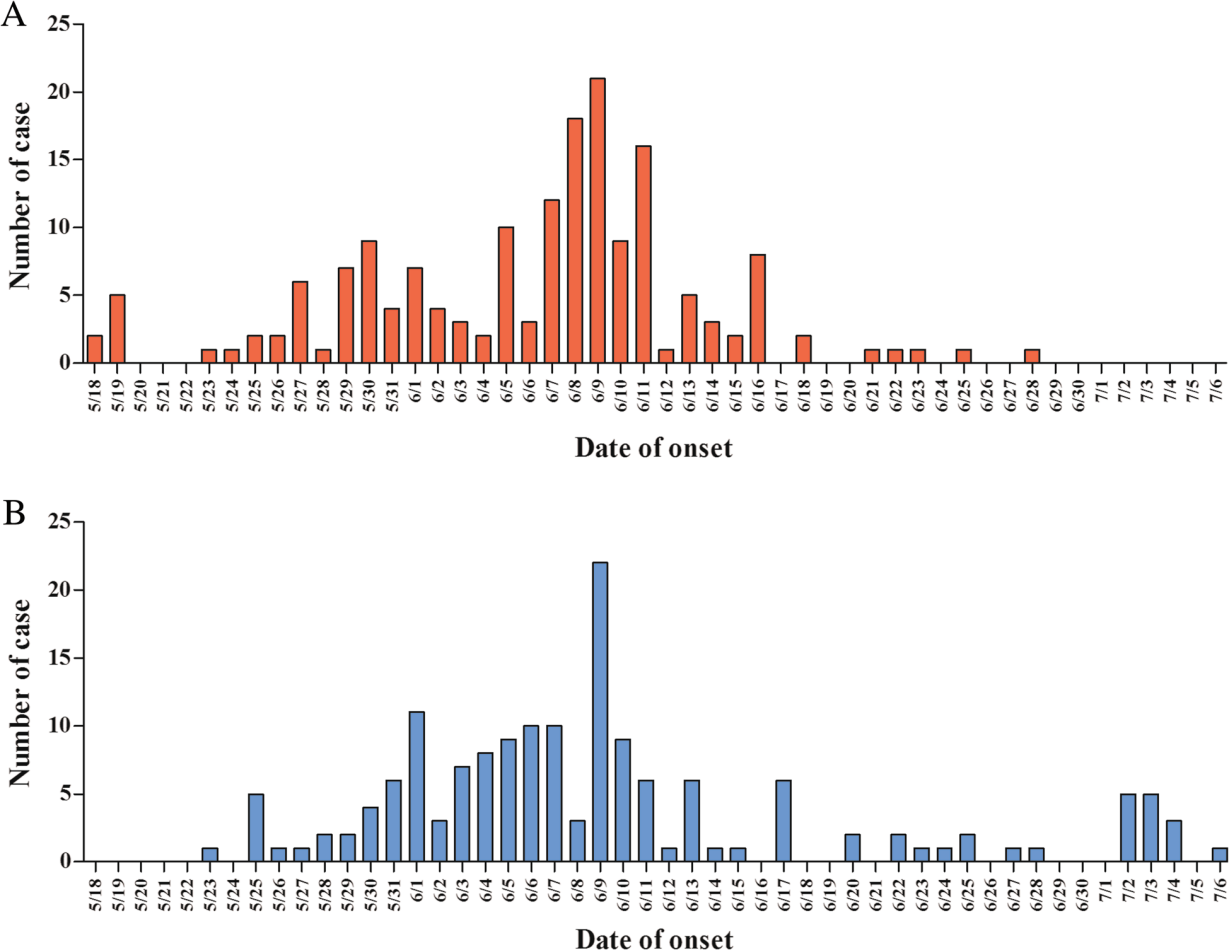
Epidemic curve of the outbreak of EKC in Weixi County Ethnic Primary School (**a**) and Yongchun Township Central Primary School (**b**)

The outbreaks began on May 18 and 23, 2018, and lasted 54 and 45 days, respectively. A total of 331 cases of acute haemorrhagic conjunctivitis (AHC) were reported; 171 cases (attack rate 30.59%, 171/559, 95%*CI*: 26.76–34.42) and 160 cases (attack rate 20.41%, 160/784, 95%*CI*: 17.58–23.24) were reported in the County Ethnic Primary School and Yongchun Township Central Primary School (Fig. 1), respectively. There was a significant difference in the attack rate between the two schools (χ^2^ = 18.217, *p* < 0.001). The average, longest, and shortest course of disease for the patients of the County Ethnic Primary School were 9.40 days, 7 days and 23 days, respectively. The numbers of male and female patients in the County Ethnic Primary School and Yongchun Township Central Primary School were 89, 82, 117 and 43, respectively. We compared the sex ratio of patients between the two schools, and the difference was statistically significant (χ^2^ = 15.626, *p* < 0.001). The median ages of the patients in the two schools were 12.34 years (range 9.91–29.53 years) and 11.00 years (range 7–37 years), respectively. Cases occurred in all grades and classes in both schools, and only one staff member in each school was ill (Table 2). According to the time distribution of the cases, both outbreaks showed human-to-human infection patterns and had two peaks with an interval of approximately 2 weeks.

**Table 2:** Final model selected for the multivariable logistic modeling of risk factors associated with infection

Variable	adj.OR	95% CI
Patient occurred in family	3.98	1.44–11.07
Contact with patient’s eye or hand	2.87	1.14–7.24
Sharing eye drop with patient	6.49	2.4–17.56
Sharing soap with patient	3.41	1.16–10.03
Sharing bedding or pillow with patient	6.18	1.49–25.72

No. of observations = 156

### Clinical manifestations

The clinical manifestations of the 160 cases included blurred vision (53.75%, 86/160, 95%*CI*: 45.94–61.60), conjunctival follicles (85.00%, 136/160, 95%*CI*: 79.41–90.59), photophobia (61.88%, 99/160, 95%*CI*: 54.27–69.48), conjunctival congestion (60.00%, 96/160, 95%*CI*: 52.33–67.67), periorbital swelling (51.89%, 83/160 95%*CI*: 44.05–59.70), eye discharge (46.25%, 74/160, 95%*CI*: 38.44–54.06), red eyes (43.13%, 69/160, 95%*CI*: 35.37–50.88), ophthalmodynia (38.75%, 62/160, 95%*CI*: 31.12–46.38), tearing (36.88%, 59/160, 95%*CI*: 29.32–44.43), pseudomembranes (22.50%, 36/160, 95%*CI*: 15.96–29.04), foreign body sensation (21.88%, 35/160, 95%*CI*: 15.40–28.35), eye itch (21.88%, 35/160, 95%*CI*: 15.40–28.35), preauricular lymph node enlargement (14.38%, 23/160, 95%*CI*: 8.88–19.87), and corneal epithelial infiltration (6.25%, 10/160, 95%*CI*: 2.46–10.04).

### Risk factors for infection

In the case-control study, 78 controls and 78 cases were interviewed. Logistic regression analysis showed that acute conjunctivitis occurrence within the family; contact with a patient’s eyes or hands or articles used by a patient; and sharing eye drops, bedding and pillows were risk factors for infection.

### Pathogen screening

A total of 38 conjunctival swabs were collected in the two outbreaks; 20 specimens were obtained from patients of the County Ethnic Primary School and 18 specimens were obtained from the Yongchun Township Central Primary School. Thirty-eight specimens were tested for chlamydia, adenoviruses and enteroviruses. Among the specimens, 26 (12 specimens from the County Ethnic Primary School and 14 specimens from Yongchun Township Central School) were positive for human adenovirus. No enteroviruses or chlamydia were detected in any of the specimens.

### Whole genome sequence and analysis

**Fig. 2:**
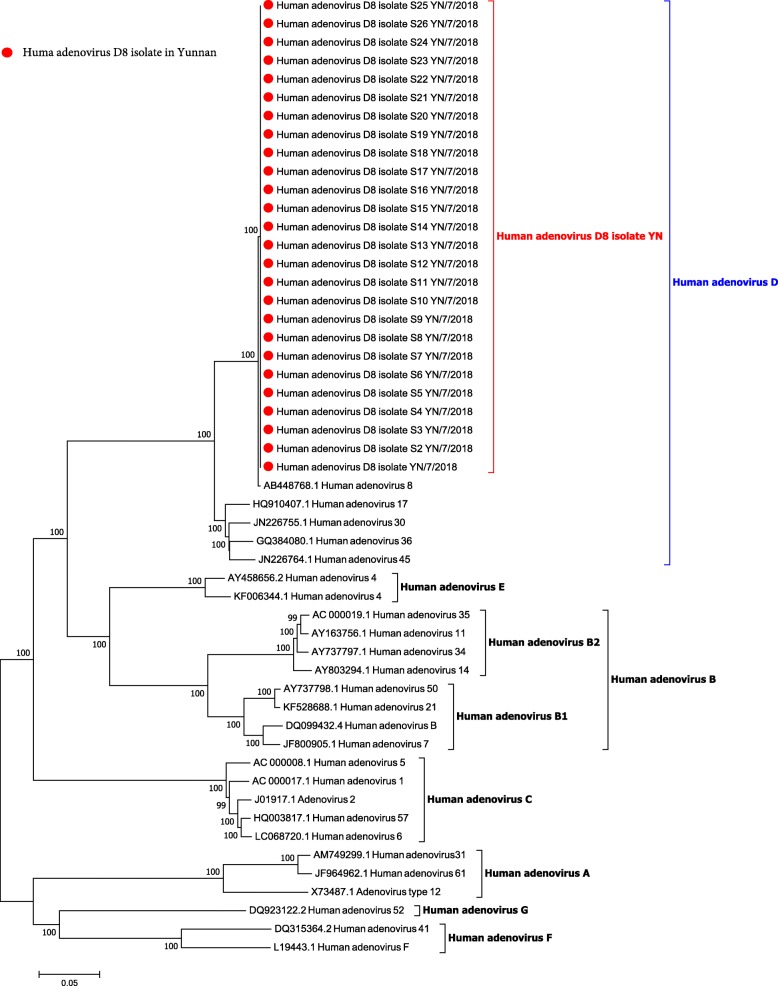
Preliminary type identification of the samples collected from the two EKC outbreaks in Yunnan in 2018, based on the whole genome of HAdV. Samples collected in Yunnan are indicated by red solid circles. Sequences of the 26 prototype HAdV strains representing the seven HAdV species (A-G) are available in the GenBank database

**Fig. 3:**
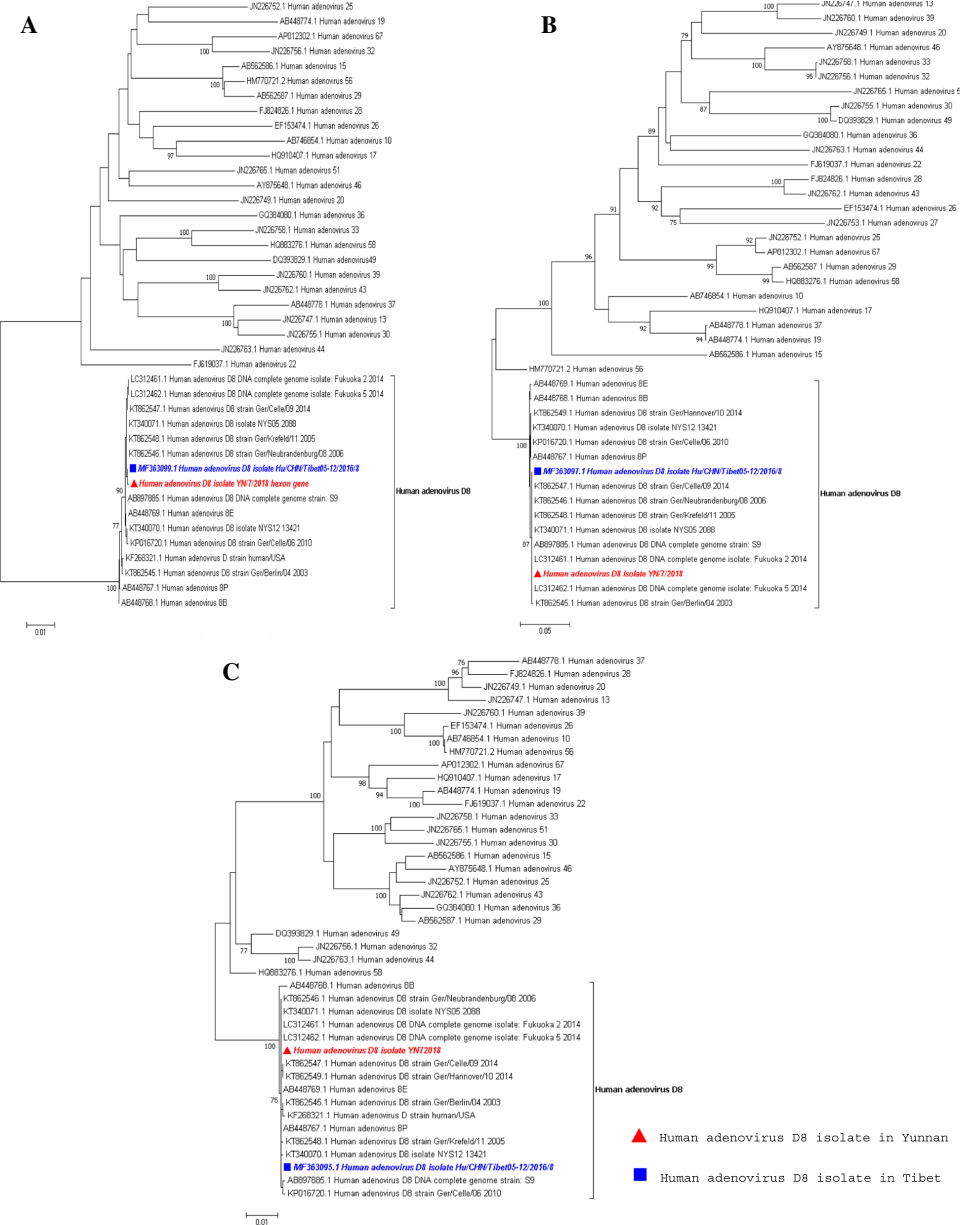
Phylogenetic analysis of the sequences of the HAdV-8 strains from the outbreaks in Yunnan compared to the HAdV-8 strains from other countries and 40 HAdV species D viruses based on **a** the entire hexon gene; **b** the entire fibre gene; and **c** the entire penton gene. The HAdV-8 strains isolated in Yunnan are highlighted in red fonts and red triangles, and the HAdV-8 strains from Tibet are indicated by blue squares

The whole genome sequence of all 26 HAdV-positive specimens was obtained by next-generation sequencing. The results of the sequence alignment indicated that the similarity of these whole genome sequences was 100%, and the viruses belonged to the same virus strain. BLAST results showed that this virus was HAdV-8. The phylogenetic tree analysis showed that 26 Yunnan HAdVs belonged to the HAdV-8 branch (Fig. 2), which was highly similar to other HAdV-8 specimens. The hexon phylogenetic tree showed that Yunnan HAdV-8 had high similarity with other HAdV 8 specimens. Notably, the genetic distance between Yunnan HAdV-8 and HAdV-8E was close and they were classified as a cluster (Fig. 3). Sequence analysis showed that the nucleotide similarity of the hexon gene between Yunnan HAdV-8 and other HAdV-8 specimens was 99.68–99.96%, with a 0–9 nucleotide differences. The amino acid similarity was 99.45–100%, with 0–3 amino acid differences. The nucleotide sequence difference in the hexon gene between Yunnan HAdV-8 and Tibetan HAdV-8 was only one base, and the nucleotide similarities of the fibre and penton genes were 100%. Moreover, the amino acid mutation caused by amino acid differences was synonymous. Yunnan HAdV-8 had the highest similarity with Tibetan HAdV-8.

## Discussion

In May 2018, two outbreaks in two boarding primary schools occurred successively, with an interval of 5 days. The epidemics lasted for approximately 2 months and one and a half months, respectively. The disease was spread widely among students in the two schools with a high incidence (30.59, 20.4%). In one of the schools, the incidence in male students was significantly higher than that in female students. The patients had typical EKC manifestations and a long course of the disease. The main risk factor for infection was close contact with the patient or articles contaminated by the patient. In the outbreak investigation, we identified an index case in Weixi Ethnic School who was infected with a propagated type and had an incubation period (IP) approximately 6 days; these characteristics fit HAdV conjunctivitis very well. The pathogen causing the outbreaks was determined to be HAdV-8. The virus was highly similar to the 2016 HAdV-8 strain in Tibet, China.

Since the 1980s, EKC outbreaks caused by HAdV-8 have been reported in different countries and regions of the world. Most of the outbreaks were caused by iatrogenic ophthalmic transmission in hospitals [25, 27, 31–37]. Two outbreaks of EKC in two schools in the Tibet autonomous region were reported for the first time in mainland China. However, the focus of this study was on virology; therefore, it lacks detailed epidemiological data [23].

This outbreak caused by HAdV-8 is the first reported in the region and the second EKC outbreak reported in mainland China. Previous studies have shown that the duration of EKC outbreaks caused by iatrogenic transmission is between 5 and 10 months, while the duration of EKC outbreaks in schools is between 1 and 1.5 months [23].

Aside from the late detection of the outbreak and the lack of timely control measures, the main reasons for the long duration of the outbreak were that the adenovirus can survive for several weeks in the environment, remain infectious on surfaces for up to a month, and resist common disinfectants [25, 34]. In addition, the patient was infectious within 14 days of the onset of symptoms [33]. Epidemiological studies of several EKC outbreaks in large ophthalmic clinics and hospitals have shown that strict hand-washing, instrument disinfection and proper medical waste disposal are not sufficient to prevent hospital transmission during an outbreak. The epidemic can only be brought under control when patients are strictly quarantined [38].

The duration of iatrogenic EKC outbreaks in hospitals is longer than that in schools. The reason may be that iatrogenic outbreaks are not easy to detect and a large number of people are exposed in batches. School outbreaks are easy to detect, and epidemic control measures can be implemented quickly. School attendance suspension may also prevent the outbreak from continuing. Most EKC outbreaks show two or more peaks, with different intervals of time between them [25, 31–34, 38]. It may be that a large number of people are exposed in groups at different times. After a long period, exposed groups will develop the disease at different times, but the exact reasons for this are unclear. According to previous studies, iatrogenic EKC outbreaks can occur throughout the year, and school EKC outbreaks occur most often between May and October. It is difficult to analyse because relatively fewer school outbreaks have occurred or been reported. Previous studies have shown that acute conjunctivitis is most common in summer [4].

The clinical manifestations, frequency of symptoms and signs of EKC and course of the disease were different from those in other studies because of variations in the virulence, sample sizes, observation time points and influence of the observer [25, 32–34, 36, 37, 39]. The patients in our study had some common manifestations of viral conjunctivitis, as well as the common characteristics of EKC, including follicular hyperplasia, pseudomembrane formation, preauricular lymphadenopathy, corneal involvement and blurred vision [14, 39–41]. Conjunctival hyperaemia and increased secretion are common features of acute conjunctivitis, while watery secretions are more characteristic of viral conjunctivitis [4].

These characteristics are of great significance for laboratory detection and field epidemiology investigations. The duration of the disease observed in this outbreak was basically consistent with that of a previous study [36]. Regarding the risk factors for transmission, previous studies have focused on risk factors for transmission in hospitals [25, 27, 31–37]. Our research reveals the risk factors for school transmission in remote areas in developing countries. Some risk factors, such as occurrence in the family, contact with a patient’s eye or hands, and sharing eye drops with a patient, are similar to those reported in previous studies. In this investigation, it was found that sharing soap, bedding, and pillows with patients were also risk factors for infection. Teachers generally have no close contact with students, so the disease is not widely spread among staff.

With the popularization and application of real-time fluorescent PCR and next-generation sequencing technologies, the detection and whole genome sequences of pathogens can be obtained in a short period of time, with practical significance for the determination and cessation of the epidemic [14, 33]. The Yunnan virus genome was highly similar to the Tibet virus gene, suggesting that the same virus strain was circulating in the region because Yunnan Diqing Prefecture is adjacent to Tibet, and the people of both localities are in close contact with each other.

Prior to the outbreak of EKC, clinical cases of AHC had been reported in the local community. The investigation of the index cases in the two schools found that the two cases came to the clinic after the onset of the disease and returned to school 3 to 4 days after the illness. These two cases may be the source of infection of the outbreak. The incubation period of the disease could not be obtained in this outbreak due to the lack of exposure time. The event was initially identified as an AHC outbreak, which was handled according to the guidelines in the diagnostic criteria and management principles for EKC [40], and the patients were isolated for 7 to 10 days. Obviously, the isolation period of 7 to 10 days for EKC patients was not enough, as some patients still shed the virus. Sometimes, it is difficult to implement strict public health measures in schools. Insufficient periods of isolation for patients and relaxed control measures may be important factors in the persistence and spread of an outbreak. In China, AHC is the only notifiable acute conjunctivitis disease. Due to the lack of an aetiological diagnosis, clinically suspected cases of viral conjunctivitis are more likely to be reported as clinically diagnosed AHC cases in many areas. There was no adenovirus isolated in our study, which may be related to specimens containing decreased virus loads.

## Conclusions

Close contact with patients or articles contaminated by patients was a risk factor for infection and further transmission of EKC. The strict isolation of patients was crucial to the control of the outbreak. Furthermore, implementing pathogen surveillance is necessary in areas with a high incidence of acute conjunctivitis.

## Data Availability

The data set used and/or analyzed during the current study is available from the corresponding author on reasonable request..

## Abbreviations

CDC: Center for Disease Control and Prevention
EKC: Epidemic keratoconjunctivitis
HAdV: Human adenoviruses
HAdv-8: Human adenovirus type 8
NGS: Next-generation sequencing
RT-PCR: Real-time polymerase chain reaction
YNCDC: Yunnan Provincial Center for Disease Control and Prevention
